# Juvenile Dysplasia Epiphysealis Hemimelica with Multiple Ankle Free Body

**DOI:** 10.1155/2021/5579684

**Published:** 2021-04-09

**Authors:** Shinsuke Sato, Song Ho Chang, Taro Kasai, Yuji Maenohara, Sho Yamazawa, Sakae Tanaka, Takumi Matsumoto

**Affiliations:** ^1^Department of Orthopedic Surgery, Faculty of Medicine, The University of Tokyo, 7-3-1 Hongo Bunkyo-ku, Tokyo 113-8655, Japan; ^2^Department of Orthopedic Surgery, Teikyo University Hospital, Mizonokuchi, 5-1-1 Niko Takatsu-ku, Kawasaki City, Kanagawa 213-8507, Japan; ^3^Department of Pathology, The University of Tokyo Hospital, 7-3-1 Hongo Bunkyo-ku, Tokyo 113-8655, Japan

## Abstract

Dysplasia epiphysealis hemimelica (DEH), also known as Trevor's disease, is a rare overgrowth of cartilage that commonly arises in the epiphyseal bone of children. We report a rare case of DEH originating from a talus accompanied by multiple intra-articular free bodies in a 7-year-old patient with ankle instability. After the primary surgery for free body removal and microfracture technique for the cartilage defects in the ankle joint, the free body recurred. Secondary surgery of arthroscopic free body removal with lateral ankle ligament repair succeeded in treating the patient, without further recurrence of the free body.

## 1. Introduction

Dysplasia epiphysealis hemimelica (DEH) is a rare asymmetrical epiphyseal cartilaginous overgrowth that is commonly seen in children or teenagers, mostly males [1]. It was originally described as a “tarsomegalie” in 1926 by Mouchet and Berlot [2]. In 1950, Trevor named this disease Trevor's disease, pointing out that the disorder originates not only from the tarsal lesion but also from other parts of the body [3]. In 1956, Fairbank renamed the condition dysplasia epiphysealis hemimelica, which is the currently used term [4]. The involvement of the affected epiphysis is hemimelic, indicating that either the medial or lateral part of the center of the ossification is affected. It contains more than one ossification centers, with varying patterns of epiphyseal chondral calcification [5]. The medial side is more common. The lower limbs are commonly affected, whereas the upper limbs and spine are rare sites [1], and dysplasia usually occurs as a single bone protuberance at the epiphysis [6]. We report a case of talus DEH accompanied by multiple free bodies in a 7-year-old patient with ankle instability, which resulted in recurrence after primary free body removal and microfracture technique to the talus lesion and obtained successful short-term results using secondary arthroscopic removal with lateral ankle ligament repair.

## 2. Case Presentation

**Figure 1:**
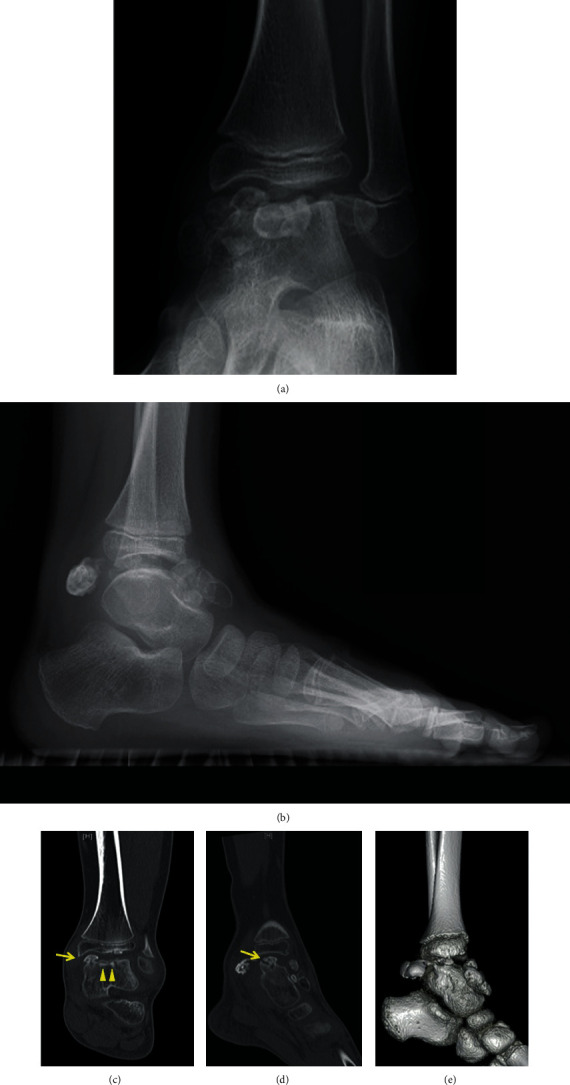
Plain radiograph: (a) anteroposterior image and (b) lateral image. Computed tomography (CT): (c) axial image, (d) sagittal image, and (e) 3-dimensional image. The left ankle plain radiograph showed multiple oval free bodies at the anterior and posterior ankle joint space. CT image showed intra-articular multiple ossified oval mass lesions (yellow arrowhead), and some protrusions arose from the talus (yellow arrow).

**Figure 2:**
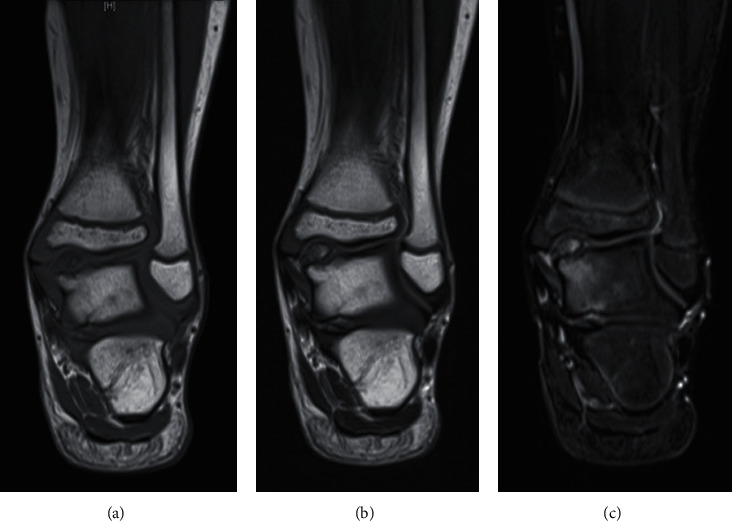
Magnetic resonance (MR) image: (a) coronal T1-weighted image, (b) T2-weighted image, and (c) short T1 inversion recovery (STIR) image. T1- and T2-weighted MR images showed an intra-articular oval mass of a low intensity partially with high intensity and with high intensity on STIR image. The diffuse STIR high-intensity lesions were confirmed on the protuberance from the talus and the area underneath it.

**Figure 3:**
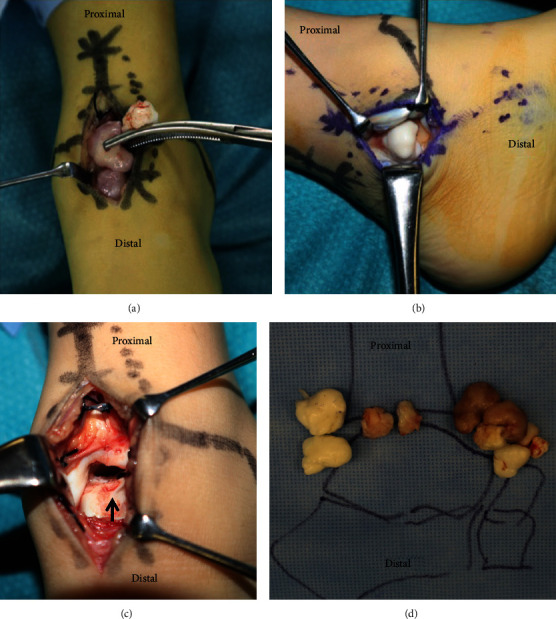
Intraoperative picture: (a) anterior midline incision, (b) posteromedial incision, (c) talar dome, and (d) removed intra-articular free bodies. Excisions of the protuberances from the talus rendered a circular articular cartilage defect (black arrow).

A 7-year-old Japanese boy presented to a nearby clinic owing to an increasing mass and pain in his left ankle for 6 months. He experienced a sprain of his left ankle several months before the onset of his symptoms. Gradually increasing pain hindered him from walking long distances and exercising. He was diagnosed as having synovial osteochondroma by a local physician and referred to our hospital. Physical examination revealed a palpable hard mass on the anterior and posteromedial sides of his left ankle. He had a full ankle range of motion of 15° in dorsiflexion and 40° in plantar flexion with his knee flexed. Instability and apprehension were evoked by an anterior drawer test on his left ankle. Left ankle plain radiography revealed multiple oval free bodies 10–15 mm in size at the anterior and posterior ankle joint spaces (Figures 1(a) and 1(b)). Computed tomography images showed intra-articular multiple ossified oval mass lesions and some protrusions arising from the talus (Figures 1(c)–1(e)). Magnetic resonance imaging showed an intra-articular oval mass with low intensity on T1-weighted images and partially high intensity on T2-weighted and short T1 inversion recovery (STIR) images. Diffuse STIR high-intensity lesions were confirmed on the protuberance from the talus and talar body underneath it, suggesting a bone marrow lesion of the protuberance and talus (Figure 2). Owing to the intractable pain and inability to walk, surgical treatment was performed. As the mass lesions were quite large to be removed under the arthroscopic procedure, open surgery was selected. With anterior midline and posteromedial incisions, intra-articular multiple mass lesions were removed (Figures 3(a) and 3(b)). Excisions of the protuberances from the talus rendered a circular articular cartilage defect 5 × 3 mm in size. The microfracture technique was performed for the cartilage defect (Figure 3(c)). No obvious mass lesion was found in the synovial tissue. As ankle instability was not the chief complaint of the patient, we did not address ankle lateral ligament repair. The wound was irrigated and closed, and a sterile dressing was applied.

**Figure 4:**
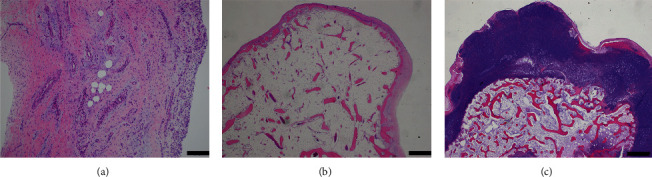
(a) Microscopic images of the synovium, (b) brown mass lesion, and (c) white mass lesion. No hyaline cartilage component was found in the synovium (a). The brown mass was trabecular bone covered by fibrous tissue (b). The white free bodies were thick cartilage cap with ossification, which resembled the epiphysis (c). Scale bar, 200 *μ*m.

On macroscopic examination, some mass lesions were colored brown, including the protuberance from the talus, while others were white (Figure 3(d)). Microscopic examination revealed no hyaline cartilage component in the synovium (Figure 4(a)). The brown mass lesion was trabecular bone tissue covered almost exclusively by fibrous tissue, and only a small amount of cartilage was found (Figure 4(b)). The white mass lesions, all of which were loose bodies, were thick hyaline cartilage (cartilage cap) with central ossification, which resembled the epiphysis (Figure 4(c)). Chondrocytic clusters were detected, and the central osseous tissue was mostly necrotic. No sarcoma component was found. As the protuberance had arisen unilaterally from the talar epiphysis of the juvenile and the synovium did not contain a cartilage component, we diagnosed it as DEH. A below-knee cast was applied for 2 weeks with non-weight-bearing for 4 weeks. Half weight-bearing was started 4 weeks after the operation, and full weight-bearing was achieved in 6 weeks. The preoperative ankle pain disappeared after the primary surgery.

**Figure 5:**
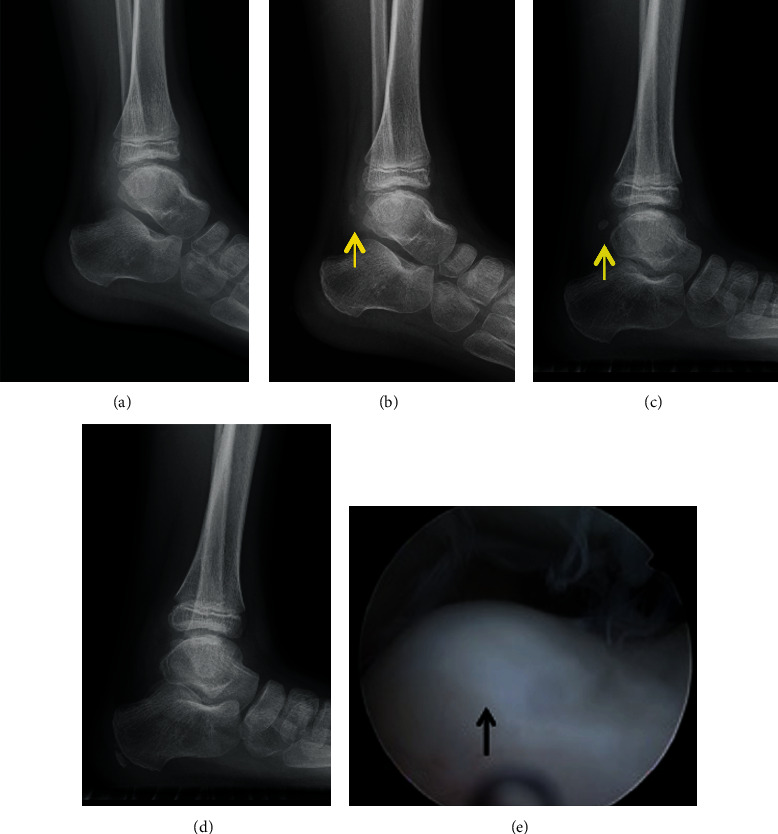
Chronological ankle lateral plain radiograph. (a) Postoperative three months after primary surgery, (b) postoperative five months after primary surgery, (c) postoperative one year after primary surgery, and (d) postoperative six months after secondary surgery. (e) Arthroscopic findings during secondary surgery. Gradual growth of a new oval ossified free body in the posterior joint space of his left ankle after primary surgery (yellow arrow) (a–c). No recurrence after secondary surgery after six months postoperatively (d). The cartilage defect due to resection of the protuberance from the talus, which was treated with microfracture technique during primary operation, was covered with healthy cartilage (black arrow).

However, 3 months after the primary surgery, the patient experienced recurrent left ankle sprain, and 10 months postoperatively, he first experienced a temporary locking of his left ankle when he plantarflexed the ankle. He had a sharp pain at the posterior side of the ankle joint during the locking position. Lateral plain radiography revealed the growth of a new oval ossified free body in the posterior joint space of his left ankle during postoperative follow-up (Figures 5(a)–5(c)). Radiographic evaluation using the anterior drawer stress test revealed a significant anterior translation of the talus compared with the contralateral side. A secondary surgery was performed to improve the patient's ankle pain and instability. As a second surgery, arthroscopic removal of the recurrent free body and anterior talofibular ligament repair were performed. Anteromedial and anterolateral portals were created for visualization of the intra-articular free body. The free body existed in the posterior portion of the ankle joint space and was removed using a grasper. The cartilage defect, resulting from the resection of the protuberance from the talus, treated with microfracture in the primary operation was covered with healthy cartilage (Figure 5(e)). The lateral ligaments were then repaired according to the modified Brostrom-Gould method using suture anchors through a longitudinal incision made over the distal fibula, which extended inferiorly toward the sinus tarsi.

Postoperatively, a below-the-knee splint was applied for 3 weeks with non-weight-bearing for 1 week. Full weight-bearing was initiated 1 week after the operation. Six months after the operation, the patient experienced neither ankle sprain nor severe ankle pain and could live a daily life without any inconvenience. A recurrence of free body or protuberance was not confirmed by the plain radiographs during the postoperative follow-up of 6 months (Figure 5(d)). We compared the outcomes of the surgery using an objective standard rating system, the Japanese Society for Surgery of the Foot (JSSF) scale [7, 8]. The preoperative JSSF scale score of 78 points (maximum score, 100 points) significantly improved to 85 points 6 months after the second surgery.

## 3. Discussion

DEH is a rare disease characterized by the overgrowth of cartilaginous tissue asymmetrically at the epiphysis of extremities. The incidence of DEH is reportedly 1 in 1,000,000 [1]. DEH normally presents as a protuberance of the epiphysis in children and young adults. Only a few cases with multiple loose bodies in the joint such as the present case have been reported in the past [6, 9] (Table 1). Histological findings of DEH include bone dysplasia with an overlying cartilage cap [1]. No malignant transformation has been reported [10]. The main candidates for differential diagnosis include osteochondroma and synovial osteochondromatosis [11] (Table 2). When DEH is fully ossified, histological findings of DEH are indistinguishable from osteochondroma [12, 13]. The location and onset of age are key to differential diagnosis [11]. Osteochondroma occurs in any bone that is preformed from cartilage; however, the most common locations are the metaphyseal region of the long bones. None of the osteochondromas are epiphyseally centered, while DEH arises from epiphysis [1]. Most patients are younger than 30 years at the time of diagnosis. Osteochondroma has an *EXT* gene mutation; however, no specific mutation has been reported on DEH [14]. We could not perform a gene analysis for *EXT* gene mutations in the present case. However, considering the localization of the tumor, we deleted the possibility of osteochondroma for the confirmed diagnosis. Synovial osteochondroma presents as a large number of osteochondral lesions originating from the synovium inside and outside of the joint. The most common age of onset is 30–60 years, and the most common locations are the knee (70%), hip (20%), shoulder, elbow, ankle, and wrist [15]. According to Evans et al., synovial osteochondroma occurred in the ankle in only 1 of 78 cases [16]. Pathological features are characterized by numerous cartilage nodules surrounded by synovial tissue. Endochondral ossification and synovial proliferation may occur [15]. The high incidence of *FN1-ACVR2A* gene fusions in synovial osteochondromatosis is reported [17, 18]. As there were multiple loose bodies in the ankle joint, synovial osteochondroma was suspected preoperatively in the present case. However, we agreed to make a confirmed diagnosis as a DEH for the present case for the following reasons: there was no hyaline cartilage in the synovium from pathological findings; synovial osteochondroma in the ankle joint of children is quite rare, and the tumor originated from the epiphysis of the talus.

To the best of our knowledge, only a few cases of intra-articular multiple DEH have been reported [6, 9] (Table 1). Only a single case of multiple loose bodies in the ankle joint has been reported in the past [9]. The possible etiology was that the brown tumor originating from the talus was the main body of DEH, and multiple loose bodies (the white mass lesions) were generated owing to the following external factors. Oates et al. proposed that osteochondral fracture may occur owing to abnormal weight-bearing stress, caused by a large bony mass arising from the surface of the talus [12]. We assume that the excessive mechanical stress stemming from the ankle instability in the present case might be an additional risk factor for this pathology. Notably, it has been reported that the free bodies in the joint may increase in size owing to synovial fluid [19].

The treatment should be individualized depending on the clinical findings. Asymptomatic lesions can be treated nonoperatively, as there are no known cases of malignant transformation [1]. Surgical treatment is usually indicated when the lesion produces pain or the deformity interferes with the joint motion [11]. Recurrence is possible if surgery is performed before the physis closure [10, 20]. In the present case, the mass lesion recurred after the primary open removal, which was successfully treated with secondary arthroscopic removal of the recurrent mass and restoration of ankle stability with lateral ligament repair. From our experience, we believe that it is important to stabilize the ankle joint in cases with ankle instability in addition to the removal of mass lesions as ankle instability can contribute to the development of recurrent free bodies. In conclusion, we encountered a rare case of DEH with multiple loose bodies in the ankle joint. Primary open removal of the mass lesion and microfracture procedure resulted in recurrence of the mass lesion. Secondary arthroscopic removal of recurrent mass lesions and ankle lateral ligament repair could obtain good short-term results.

## Figures and Tables

**Table 1:** Key findings of published work of multiple loose body of DEH.

Reference	Type of study	Age	Gender	Affected site	Treatment	Follow-up
Wheeldon and Altiok [6]	Case report	9 years old	Male	Knee	Loose body removal	6 weeks
Calderaro et al. [9]	Case report	10 years old	Male	Ankle	Loose body removal	5 years

**Table 2:** Differential diagnosis of DEH.

	DEH	Osteochondroma	Synovial osteochondromatosis
Onset of age	Children and young adults	Younger than 30 years	30–60 years
Location	Epiphysis	Metaphyseal region of the long bones	Inside and outside of the joint
Histological findings	Bone dysplasia with an overlying cartilage cap		Numerous cartilage nodules surrounded by synovial tissue
Genetic alterations	Not reported	*EXT* gene	*FN1-ACVR2A* fusion

## Data Availability

The data used to support the findings of this study are included within the article.
